# Positive associations between galectin-3 and PSA levels in prostate cancer patients: a prospective clinical study-I

**DOI:** 10.18632/oncotarget.12619

**Published:** 2016-10-12

**Authors:** Kosei Nakajima, Lance K Heilbrun, Victor Hogan, Daryn Smith, Elisabeth Heath, Avraham Raz

**Affiliations:** ^1^ Department of Oncology, Karmanos Cancer Institute, School of Medicine, Wayne State University, Detroit, Michigan, USA; ^2^ Department of Pathology, Karmanos Cancer Institute, School of Medicine, Wayne State University, Detroit, Michigan, USA; ^3^ Biostatistics Core, Karmanos Cancer Institute, Wayne State University, Detroit, Michigan, USA

**Keywords:** galectin-3, PSA, autoantibody, diagnostic biomarker, prostate cancer

## Abstract

Galectin-3 (Gal-3), an oncogenic pro-inflammatory protein, has been suggested as a possible complementary diagnostic candidate to prostate specific antigen (PSA) blood test for prostate cancer patients. The presence of the proteins in the circulation (biomarkers) may elicit an intrinsic humoral immune reaction by generating autoantibodies, which consequently could alter the detection levels. Here, we report the associations of the two prostate cancer biomarkers, Gal-3 and PSA in patients at different clinical states of prostate cancer while taking into account the autoantibody levels. A blind, prospective, single institution, pilot study was conducted. A total of 95 men were classified into 5 groups: healthy controls (Group1), newly diagnosed patients (Group2), no recurrence after local therapy (Group3), rising PSA after local therapy (Group4), and metastatic patients (Group5). Gal-3 and PSA level were divided by their respective autoantibodies, which yielded relative PSA and relative Gal-3 levels. After the adjustments, Spearman's rank correlations and linear regression modeling revealed the positive associations between relative Gal-3 and relative PSA levels among all 95 men combined (rho = 0.446, *P* < 0.0001; fitted slope 0.448, *P* < 0.0001), in Group2 (rho = 0.616, *P* = 0.0050; fitted slope 0.438, *P* =0.0011), and Group3 (rho = 0.484, *P* = 0.0360; fitted slope 0.470, *P* = 0.0187). The data show positive associations of relative Gal-3 and relative PSA levels in prostate cancer patients, notably at early clinical time course. Allowing for the influence of autoantibodies, Gal-3 level might be considered as a potential biomarker since it is positively associated with PSA level.

## INTRODUCTION

The American Cancer Society has estimated that there will be 180,890 new diagnoses and 26,120 deaths of prostate cancer in the USA in 2016. To successfully screen for the disease in a wide-ranging population of men, a simple and reliable diagnostic method is crucial. Most prostate cancers are first diagnosed by digital rectal examination and an abnormal prostate-specific antigen (PSA) level in the blood. However, PSA tests have shortcomings; false-positive (higher PSA without cancer) and false-negative (lower PSA despite cancer) have been frequently reported. Consequently, the U.S. Preventive Services Task Force recommends against PSA-based screening for prostate cancer [1]. It is of paramount need to understand and overcome factors that impact PSA results. As a possible cause, one study showed that both healthy men and prostate cancer patients intrinsically harbor antibodies to PSA as a self-antigen, *i.e.* PSA autoantibody (AAPSA), reducing PSA concentration. The finding suggests that the cases of lower PSA despite prostate cancer progression or higher PSA without prostate cancer may be caused by the autoantibody. Thus, autoantibody adjustments may be indispensable when viewing/interpreting PSA levels [2].

To complement the PSA test, another focus was to examine the diagnostic efficacy of Galectin-3 (Gal-3), a lectin-family oncogenic protein expressing in both primary and secondary lesions of prostate cancer. Extracellular Gal-3 contributes to cancer proliferation, chemotherapeutic resistance, angiogenesis, endothelial adhesion to the distant organs, and metastatic bone destruction throughout prostate cancer progression [3–9]. Thus, Gal-3 is closely associated with malignancies, considerably influencing local microenvironments, which results in debilitation of cancer patients. In systemic circulation, elevated Gal-3 serum level was reported in metastatic prostate cancer patients in a pre-pilot study, implying a possible complementary diagnostic marker together with the PSA test [10]. A better understanding of the relationship of Gal-3 and PSA, in patients at different states of prostate cancer, is needed prior to clinical application.

The purposes of this study were, while incorporating the influence of autoantibodies, 1- to determine Gal-3 levels, and 2- to examine the relationship between PSA and Gal-3 along with the clinical status of the patients enrolled.

## RESULTS

### Positive association between relative Gal-3 and relative PSA among all 95 men

**Figure 1:**
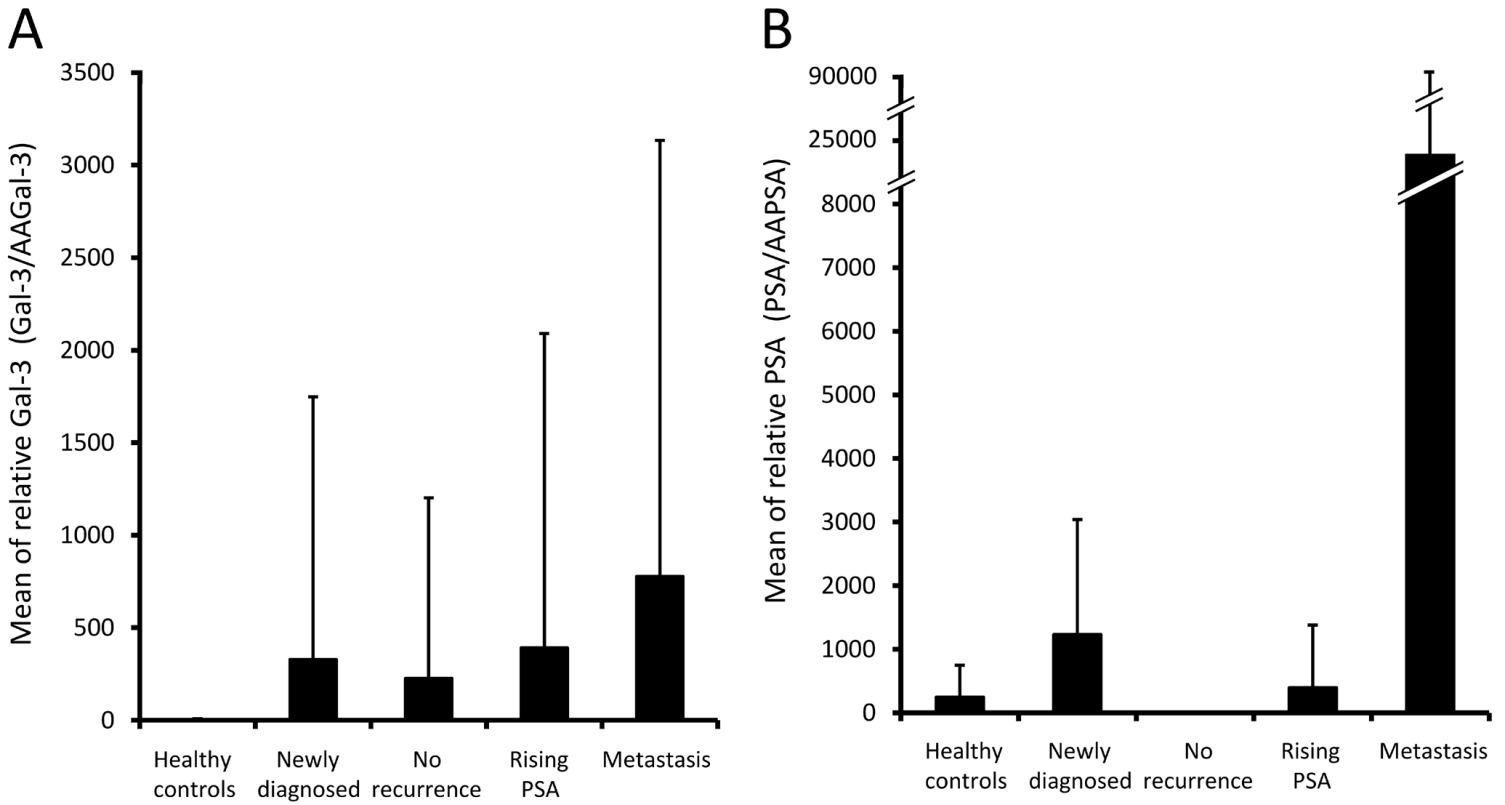
Relative Gal-3 and relative PSA levels in patients at different clinical states of prostate cancer Gal-3 and PSA concentrations were divided by their respective autoantibody levels, and the values were referred to as relative Gal-3 and relative PSA. Bars show the mean values of **A.** relative Gal-3 and **B.** relative PSA Whisker heights indicate standard deviations.

A masked, prospective, single institution, pilot study was planned. A total of 95 participants was classified into 1 of 5 groups: healthy controls with no history of current invasive cancer (Group 1); newly diagnosed patients with intact prostate cancer (Group 2); patients who had no evidence of disease recurrence post local therapy (Group 3); patients with rising PSA after local therapy (Group 4); or patients with metastatic prostate cancer (Group 5). The expression levels of PSA, AAPSA, Gal-3, and AAGal-3 were determined in both healthy controls and prostate cancer patients [2]. Based on the hypothesis that autoantibodies alter the levels of cancer-related antigens, the PSA and Gal-3 concentrations were divided by their respective autoantibody level so as to create a relative measure of each antigen (*i.e.* ratio) rather than their absolute measures. It also yielded a measure of the amount of antigen per unit of respective autoantibody. The divided values were referred to as relative PSA and relative Gal-3. Table 1 shows descriptive statistics after the adjustment. Briefly, the means of relative Gal-3 were (Group1) 3.0, (Group2) 327.4, (Group3) 225.5, (Group4) 391.5, (Group5) 776.5 (Figure 1A). The means of relative PSA were (Group1) 244.3, (Group2) 1230, (Group3) 3.3, (Group4) 395.1, (Group5) 23682 (Figure 1B). The variations in these mean values suggested that the relative Gal-3 values respond to the presence of cancer or clinical classifications, similarly to relative PSA or to original PSA levels. However, there was no significant difference in the relative Gal-3 levels across the five clinical groups (*P* = 0.1499).

**Table 1:** Summary statistics of relative Gal-3 and relative PSA levels

Group	Relative variable	N	Mean	Standard deviation	Median	Minimum	Maximum
							
All men	PSA	95	5111	31078	2.2	0.0	289375
	Gal-3	95	344.8	1500	1.3	0.3	8671
							
Healthy controls	PSA	19	244.3	503.1	0.8	0.1	1667
	Gal-3	19	3.0	4.7	1.4	0.4	17.2
							
Newly diagnosed	PSA	19	1230	1810	7.9	0.1	5542
	Gal-3	19	327.4	1420	0.9	0.3	6192
							
No recurrence	PSA	19	3.3	7.8	0.0	0.0	20.8
	Gal-3	19	225.5	976.6	0.8	0.4	4258
							
Rising PSA	PSA	19	395.1	984.2	3.2	0.1	3333
	Gal-3	19	391.5	1698	1.3	0.7	7404
							
Metastasis	PSA	19	23682	67701	14.4	0.0	289375
	Gal-3	19	776.5	2357	2.1	0.3	8671
							

Gal-3 and PSA concentrations were divided by their respective autoantibody levels, and the values were referred to as relative Gal-3 and relative PSA. Descriptive statistics of the relative Gal-3 and relative PSA level are shown separately for each clinical classification.

Next, the association between relative Gal-3 and relative PSA was analyzed. For all 95 men combined, the rank correlation of relative PSA with relative Gal-3 was rho = 0.446, which was highly significantly different from zero (*P* < 0.0001) [Table 2, upper left]. Further, linear regression modeling of relative PSA and relative Gal-3 was performed, and the relationship was visually examined. Ten different transformations were applied to relative PSA and relative Gal-3. Only the rank transformation adequately Normalized the distribution of both variables. The regression modeling showed that the fitted slope for rank (relative PSA) and rank (relative Gal-3) was positive (0.448) and highly statistically significant (*P* < 0.0001). The fitted regression model was: rank (relative PSA) = 26.517 + 0.448*rank (relative Gal-3) [Table 2, upper right]. The models had no identified leverage points. Hence, there was no need for a sensitivity analysis. In addition, no autoantibody covariate adjustment was possible since all 4 biomarkers were utilized in the creation of these autoantibody-adjusted measures of PSA and Gal-3. Taken together, there was a highly statistically significant positive association of relative PSA with relative Gal-3 among all 95 men combined.

**Table 2:** Association statistics between relative Gal-3 and relative PSA levels

Group	rho	*P*-value	Slope	90% CI	*P*-value
All men	0.446	< 0.0001*	0.448	(0.302, 0.593)	< 0.0001*
Healthy controls	0.347	0.1451	0.347	(0.031, 0.664)	0.0734
Newly diagnosed	0.616	0.0050*	0.438	(0.244, 0.633)	0.0011*
No recurrence	0.484	0.0360*	0.470	(0.156, 0.785)	0.0187*
Rising PSA	0.202	0.4075	0.162	(-0.048, 0.372)	0.1967
Metastasis	0.395	0.0944	0.368	(-0.074, 0.810)	0.1661

Gal-3 and PSA concentrations were divided by their respective autoantibody levels, and the values were referred to as relative Gal-3 and relative PSA. The table shows Spearman rank correlation coefficients (rho values) and their *P*-values (left) between relative Gal-3 and relative PSA level for each clinical group. The fitted slope, its 90% confidence interval (CI), and *P*-value are also shown for the linear regression model of relative PSA and relative Gal-3 (right). A *P*-value of less than 0.05 was considered statistically significant and shown with asterisks (*).

### Significance of relative Gal-3 level in the clinical course of prostate cancer

The finding above prompted further statistical evaluation within each clinical classification separately. Table 2 (lower left) summarizes the Spearman correlation coefficients. The results showed that all 5 rho values were positive; 2 of them were statistically significantly different from zero, *i.e.* rho = 0.616, *P* = 0.0050 in newly diagnosed patients, and rho = 0.484, *P* = 0.0360 in patients with no recurrence.

Further, the relationships were examined by linear regression modeling separately for clinical classification [Table 2, lower right]. For relative Gal-3, only the rank transform yielded approximate Normality in all 5 Groups. For relative PSA, the natural logarithm (ln) transform yielded approximate Normality of the ln(relative PSA) distributions for Groups 2 (Newly diagnosed), Group4 (Rising PSA), and Group5 (Metastasis). However, ln(relative PSA) was still highly significantly non-Normal for Group 1 (Healthy controls) and Group 3 (No recurrence). Only the rank transform yielded approximate Normality of the relative PSA distribution for these two Groups. The univariate linear regression model did not need to be extended to a bivariate model by including either autoantibody variable because the effect has already been taken into account in the creation of the relative (adjusted) measure of each antigen. With those mandatory prerequisite steps accomplished, then linear regression modeling was performed.

For Group 1 (Healthy controls), the fitted slope (0.347) resulted from a weak positive relationship (*P* = 0.0734), and there were no leverage points.

For Group 2 (Newly diagnosed), the result showed an overall positive and statistically significant fitted slope (0.438) with *P* = 0.0011. The one ln(relative PSA) slight outlier was still well within the rank(relative Gal-3) distribution and was not a leverage point unduly influencing the estimate of the slope.

For Group 3 (No recurrence), the analysis showed an overall positive and statistically significant fitted slope (0.470) with *P* = 0.0187. The one rank(relative PSA) slight outlier was nearly centered within the rank(relative Gal-3) distribution and was not a leverage point unduly influencing the estimate of the slope.

For Group 4 (Rising PSA), the results showed an overall slightly positive, but not statistically significant fitted slope (0.162) with *P* = 0.1967, and there were no leverage points.

For Group 5 (Metastasis), the fitted slope (0.368) was positive, but not statistically significant (*P* = 0.1661). The one rank (relative PSA) slight outlier was still within the rank (relative Gal-3) distribution and was not a leverage point unduly influencing the estimate of the slope.

**Figure 2:**
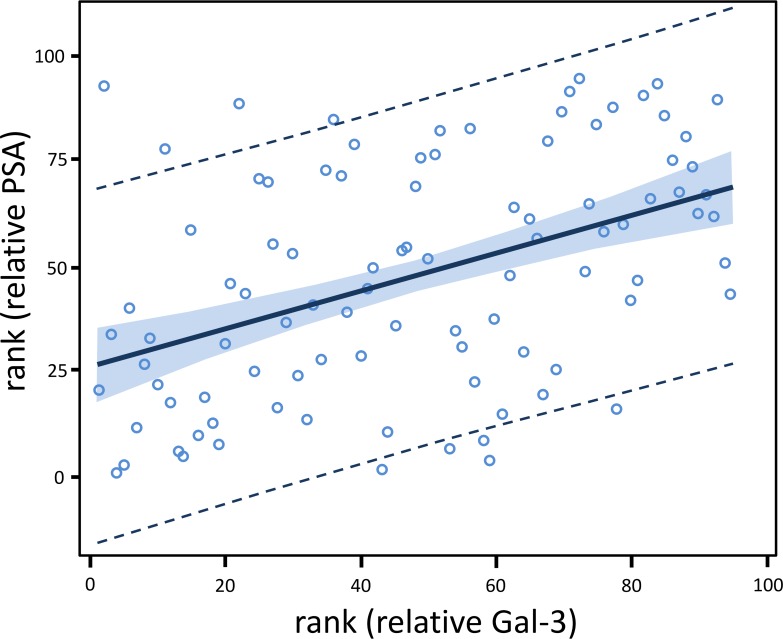
Relative Gal-3 level is positively associated with relative PSA level among all 95 men Gal-3 and PSA concentrations were each divided by their respective autoantibody level, and the values were referred to as relative Gal-3 and relative PSA. The fitted regression model was: rank (relative PSA) = 26.517 + 0.448*rank (relative Gal-3) (*P* < 0.0001). The lines at the outer edges of the blue band define the 90% confidence limits (CLs) for predicting the mean of rank (relative PSA) for a given value of rank (relative Gal-3). The dashed lines define the 90% confidence limits (CLs) for predicting an individual value of rank (relative PSA) for a given value of rank (relative Gal-3).

## DISCUSSION

The present study revealed that, after adjusting for autoantibody levels, Gal-3 levels are positively associated with PSA concentration. The similar behavior in systemic circulation suggests that the relative Gal-3 might be an additional maker for prostate cancer. Specifically, the relative Gal-3 level might need to be considered during diagnostic screening and disease recurrence monitoring. The cause of a potentially positive association of the two biomarkers may originate from a common feature that Gal-3 and PSA both have been reported to reflect on cancer malignancy [9, 11]. In contrast, the two are expressed in different cellular origins of prostatic epithelial structure; PSA expresses in luminal-oriented prostate cancer [12], whereas Gal-3 expresses in basal cell-origin [13]. Further, their secretory mechanisms are also different [9, 14, 15]. In light of this similarity and dissimilarity, in addition to a PSA test, Gal-3 monitoring could help detect wide-ranging, heterogeneous characteristics of prostate cancer cells as a part of comprehensive diagnosis while explaining malignancy.

Our finding clarified the importance of autoantibody adjustment when considering the association of the cancer biomarkers Gal-3 and PSA. In the current study, we used simple division to incorporate the influence of autoantibodies. A different mathematical formula and/or a larger number of samples may improve our ability to distinguish among clinical groups.

In conclusion, the data largely support the original report [10], highlighting a potential association between PSA and Gal-3 levels in blood. In addition, there is a probable need to adjust PSA test results routinely in the clinic due to the presence of AAPSA [2]. Further, we hypothesize the need for an adjusted Gal-3 level that is affected by the AAGal-3 generation in response, and should be considered as a factor in cancer and inflammatory diseases.

## MATERIALS AND METHODS

### Study patients

Eligible men were age ≥ 18 (if already diagnosed with prostate cancer). Previous history of chemotherapy may possibly confound AA levels because in general, the treatment suppresses the immune function. Such patients were not included in this study. To classify prostate cancer patients, they were examined by PSA, trans-rectal ultrasound (TRUS), and prostatic biopsy. Metastatic lesions were detected by chest X-rays, CT, MRI, bone scan, and/or F-18 sodium fluoride positron emission tomography (NaF-PET). From October 2013 to July 2015, patients were recruited from genitourinary oncology clinics, Karmanos Cancer Institute, and then gave informed consent to be participants. The study-related information of patients was recorded in the Online Collaborative Research Environment (OnCore^®^) database. Patients’ whole bloods were collected using two 5 ml serum separator tubes, and were centrifuged at 5000 rpm for 5 minutes to separate serum from cellular components. The serums were aseptically transferred to cryovials labeled with limited information in a safety cabinet, and then frozen at −80°C. Due to the need for unbiased assays, the clinical information (*i.e*., patient Group identification) was masked to the laboratory investigators.

### ELISA

Customized ELISA plates were generated to detect AAGal-3 and AAPSA contained in prostate patients’ sera. First, human recombinant Gal-3 [7] or PSA (Novus Biologicals, CO) were diluted by 100nM bicarbonate/carbonate coating buffer (pH9.6). Then, 94ng of recombinant Gal-3 and 10ng of recombinant PSA were incubated in each well of Nunc-immuno^TM^ MicroWell 96 well solid plates (Thermo scientific, Waltham, MA) for 1 hour at 37°C. Simultaneously, human normal IgG (Invitrogen, Carlsbad, CA) was serially diluted and incubated on the plate for the standard curve. After fixation of the proteins, the liquids were discarded. The wells were washed 4-times using TBS with tween 20 (0.1%). Blocking was performed using 1% BSA/coating buffer for 1 hour at 37°C. Patient's sera were diluted 80-fold using phosphate buffered saline (PBS) with 0.75% BSA plus 0.1% tween 20 for AAPSA detection. For AAGal-3 detection, the sera were diluted 160-fold using PBS with 1% BSA plus 0.5% tween 20. Then, 100ul of diluted sera were incubated for 1 hour at 37°C. After washing, anti-human IgG peroxidase-conjugated antibodies (Rockland, PA) were reacted for 1 hour at 37°C. Then, tetra-methyl-benzidine (TMB), a substrate for peroxidase, was incubated for 20 min at room temperature. The enzymatic reactions were terminated by addition of 0.5M sulfonic acid. Absorbance was measured at 450nm. In order to eliminate non-specific reactions, wells without recombinant proteins were also prepared, and incubated with each patient's serum. The net absorbance was calculated as following formula: (absorbance with recombinant protein) - (absorbance without recombinant protein). Then, concentration was determined by extrapolation into the standard curve, whereby the range of 4.8 - 312.5 ng/ml was measurable. As for Gal-3 concentration, Galectin-3 ELISA kit (BG Medicine, Waltham, MA) was used. The measurements were also performed in duplicate following the manufacture's protocol. The mean of the duplicates was used in all statistical analyses. An ELISA plate stratified randomization procedure was used to assign patients’ samples to wells for each plate so as to minimize confounding due to plate effects, row effects, or column effects. The original values were available on clinical study-II [2]. Then, the determined PSA and Gal-3 concentrations were divided by their respective autoantibody levels to create a relative measure of each antigen (*i.e.* ratio). The divided values were referred to as relative PSA and relative Gal-3.

### Statistical methods

#### Design

The objective was to identify Gal-3, PSA, AAGal-3, and AAPSA in the serum of men in 5 different states of prostate cancer. The primary statistical endpoints were the levels of each of those 4 study biomarkers. Within each group of men, it was desired to estimate the mean biomarker level to within 0.40 standard deviations (SD's) of the true mean, with 90% confidence. The study required N = 19 men per group, hence 19*5 = 95 patients in total. The required sample size per group was determined *via* the ‘Confidence Intervals for One Mean’ program in the Power And Sample Size (PASS) 11 software [16].

#### Analysis

For all 95 men, and separately for each group, and the relative Gal-3 and relative PSA levels were summarized with standard descriptive statistics, number of each group (N), mean, standard deviation (SD), median, minimum value, and maximum value. The nonparametric Kruskal-Wallis test was used to compare a given biomarker across clinical groups. To first evaluate the association between any pair of continuous variables, the nonparametric Spearman's rank correlation coefficient was calculated to obtain a provisional indication of the direction and strength of linear association. To characterize the statistical relationship between relative Gal-3 and relative PSA, ordinary least squares (OLS) linear regression modeling was used. Normality testing of the study variables was performed separately within each of the 5 clinical groups, and for all 95 men combined. Ten transformations were generated (null [no transform], ln, log_10_, square root, cube root, fourth root, fifth root, inverse, inverse squared, and rank), and tested for Normality. Four tests of Normality were performed: Shapiro-Wilk, Kolmogorov-Smirnov, Cramer-vonMises, and Anderson-Darling. Non-Normality was concluded if at least 2 of those 4 tests were significant at the 0.01 alpha level. If more than 1 transform Normalized a given study variable, the transform that was the mathematically simplest was chosen. Then, linear regression modeling was performed using transformed variables. Model residuals were thoroughly examined to assess goodness of fit. Sensitivity analyses were also conducted after excluding leverage points identified in the regression models. The SAS software version 9.4 (SAS Institute, Cary, NC) were used for statistical analyses. All tests of statistical significance were two-sided. A *P* value of less than 0.05 was considered statistically significant. Given the pilot nature of the study, no adjustments were made for multiple comparisons.
